# Tracing nitrate sources with dual isotopes and long term monitoring of nitrogen species in the Yellow River, China

**DOI:** 10.1038/s41598-017-08756-7

**Published:** 2017-08-17

**Authors:** Fu-Jun Yue, Si-Liang Li, Cong-Qiang Liu, Zhi-Qi Zhao, Hu Ding

**Affiliations:** 1 State Key Laboratory of Environmental Geochemistry, Institute of Geochemistry, Chinese Academy of Sciences, Guiyang, 550081 China; 2 School of Geographical and Earth Sciences, University of Glasgow, Glasgow, G12 8QQ United Kingdom; 3 Institute of Surface-Earth System Science, Tianjin University, Tianjin, 300072 China; 4 State Key laboratory of Hydraulic Engineering Simulation and Safety, Tianjin University, Tianjin, 300072 China

## Abstract

A heavy load of nitrogenous compounds reflects nutrient loss and influences water quality in large rivers. Nitrogenous concentrations and dual isotopes of nitrate were measured to ascertain the spatial and temporal distributions of nitrate transformation in the Yellow River, the second-longest river in China. Assessment of the long-term record indicates that [NO_3_^−^–N] has increased by two-fold over the past three decades. Weekly observation of ammonium over a twelve-year period revealed high concentrations and suggests impairment of water quality, particularly since 2011. The estimated total dissolved nitrogen flux was 7.2 times higher in middle reaches than that at head waters. Anthropogenic nitrogen sources become more important in lower section of the upper reaches and middle reaches because of intensive agricultural activities and urban input. Nitrate in the lower reaches was mainly derived from transportation of upstream nitrate and point sources from cities. The spatial variation of ammonium and nitrate isotopes show that nitrification is a key process governing nitrogen transformation. Riverine biological processes could potentially be responsible for the shift of nitrate isotope signature. The first step to reducing nitrogen load and improving water quality will be containment and careful management of sources from urban input, sewage waste and irrigation runoff.

## Introduction

Elevated concentrations of nitrogenous compounds in riverine environments is well demonstrated as having detrimental consequences for human and ecosystem health^1, 2^. Long-term monitoring is crucial to understanding nitrogen (N) dynamics and transformation in rivers. Over the past 50 years, the nitrogen load in watersheds has been heavily impacted by urbanization and land-use practices associated with intensification of agriculture and animal farming^3, 4^. N export to the sea by large rivers has increased significantly during the past five decades, with up to a six-fold increase in N fluxes caused by human activity observed in the United States^2, 5, 6^.

Nitrate and ammonium are the two dominant forms of reactive N entering the aquatic environment. The major sources of nitrate are atmospheric deposition, chemical fertilizers, soil organic nitrogen (SON), manure, and sewage effluents^7^. The nitrogen cycle in riverine systems involves several transformations, primarily nitrification, denitrification and biological uptake^7, 8^. Elucidation of the sources of nitrogen and riverine transformations that most influence N export is essential for understanding the sustainability of freshwater systems and environmental changes on the Earth’s surface^4, 8^. Paired stable nitrogen and oxygen isotopes are a powerful tool that is highly suitable for this research objective^7–13^.

**Figure 1:**
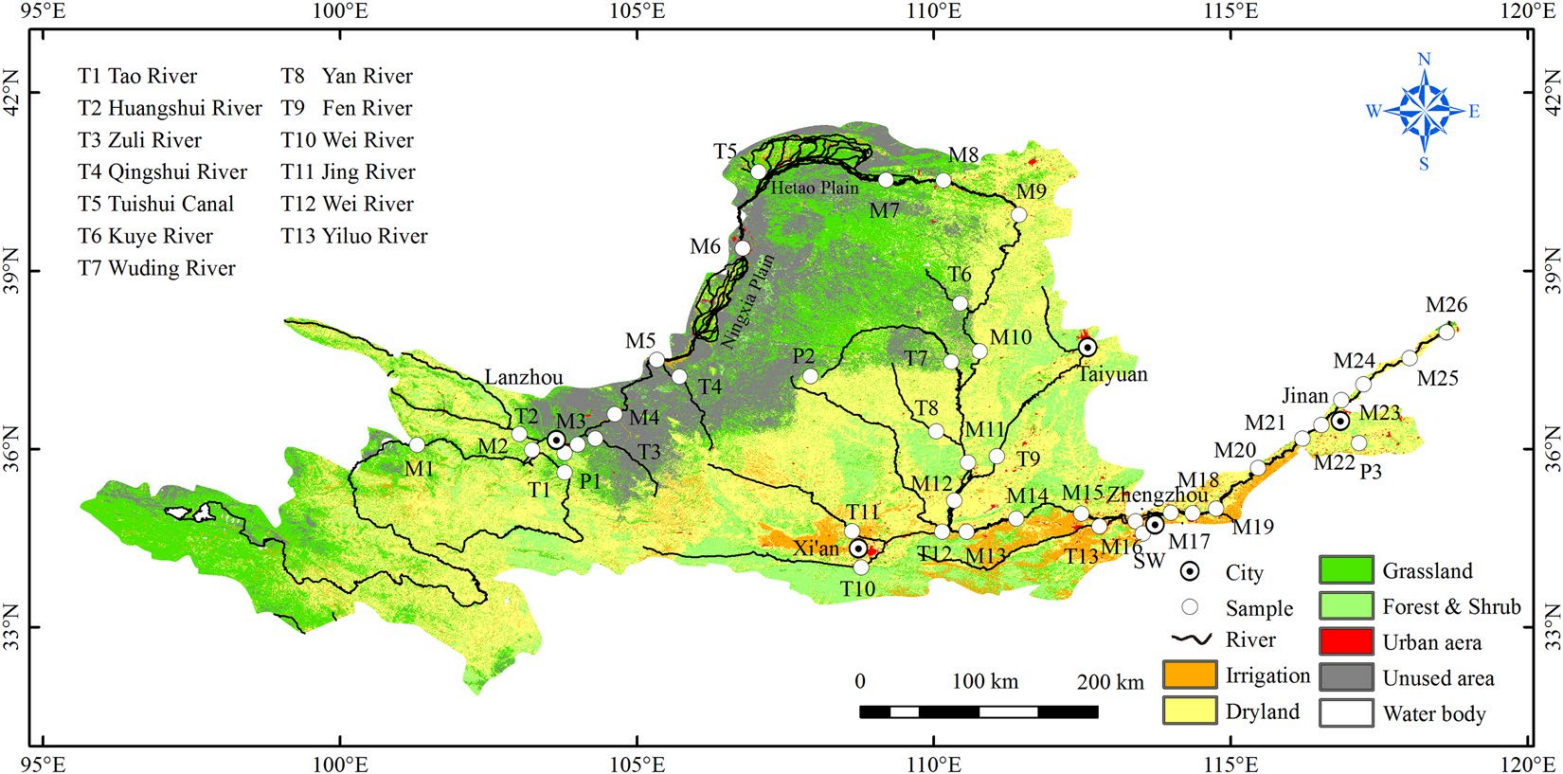
Land use of Yellow River Basin and sampling sites where M indicates samples collected from the mainstream; T indicates samples collected from tributaries; P indicates precipitation samples and SW indicates sewage waste). Map produced using ArcGIS software 9.3 (http://www.arcgis.com/features/index.html).

The Yellow River (YR) is the second longest rivers in China, with drainage area of 7.95 × 10^5^ km^2^ and an annual average discharge of 5.8 × 10^11^ m^3^ and so is a significant drainage system globally. The Yellow River provides 140 million people with drinking and irrigation water in regions where the water supply per person is less than one third of the national average and water quality has suffered greatly from pollution^14^. Few long-term studies of riverine nitrogen in the YR have been conducted, and even fewer studies have used the dual isotopes of nitrate to identify its sources and behaviors^15–20^. In this study, samples representing the whole watershed were collected across the wet and dry seasons (Fig. 1) to understand the spatial variations in nitrogen speciation and concentration based on water chemistry and nitrate isotopic composition. Three representative sites were then identified from the main channel of YR for monthly sampling to further constrain temporal variation. These three sites are M1 in the headwaters with a relatively natural drainage area, M17 in the middle reaches, constituting 97% of the entire basin area and M23 in the lower reaches, respectively. The riverbed in the lower reaches is above ground level due to the combined effect of levee bank construction and sediment deposition from the highly sediment laden waters discharged from the middle reaches.

## Results

### Seasonal variation in concentration of nitrogen compounds and chloride

The concentration of total dissolved nitrogen ([TDN]) ranged from 0.53 to 4.1 mg·L^−1^ (mean ± 1 SD = 2.6 ± 1.0 mg ·L^−1^, n = 26) in the main channel (in future referred to as mainstream) during the high flow season (Figure S1), which is lower than [TDN] in the low flow season (4.7 to 8.5 mg·L^−1^ and n = 16, mean = 6.0 ± 1.1 mg·L^−1^) (one-tailed t-test, p < 0.0001, α = 0.05). The [TDN] in tributaries ranged from 0.54 to 12.0 mg·L^−1^ with a mean value of 4.3 ± 3.6 mg·L^−1^ (n = 13) during the high flow season (Figure S1), and 7.0 to 33.5 mg·L^−1^ (n = 6) in the low flow season.

**Figure 2:**
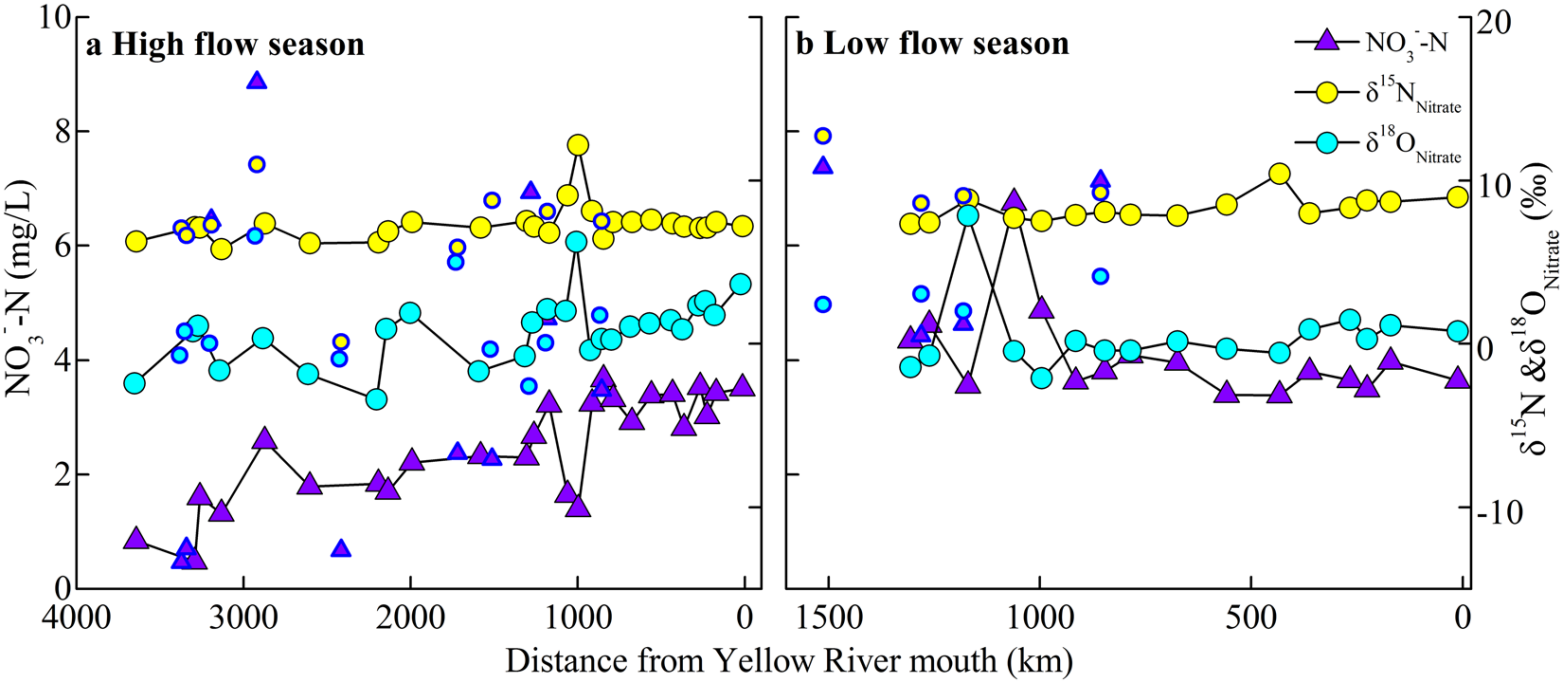
The distance variation of [NO_3_^−^–N] and nitrate isotopes in Yellow River, scatter symbol indicates tributary samples.

Nitrate was the dominant nitrogenous compound during both two flow seasons (Figure S1). [NO_3_^−^–N] from main stream in the high flow season were generally lower than that in the low flow season (Fig. 2). The average [NO_3_^−^–N] of 2.5 ± 0.9 mg·L^−1^ in the mainstream was higher than that of the two other major rivers (Songhua River 1.06 ± 0.69 mg·L^−1^and the Changjiang River 0.78 ± 0.3 mg·L^−1^) in China during the high flow season^10, 21^. All mainstream samples exhibited [NH_4_^+^–N], dissolved organic nitrogen concentration ([DON]) and [NO_2_^−^–N] of less than 0.5 mg·L^−1^ (Figure S1). Three tributary samples (T6, T9 and T10) had [NH_4_^+^–N] of greater than 2.0 mg·L^−1^. The [NH_4_^+^–N] and [DON] relatively increased relative to other compounds during the low flow season, particularly DON in the mainstream samples. The highest [NH_4_^+^–N] and [NO_2_^−^–N] was observed at T9 (Fen River) during both flow seasons, and the highest [DON] was observed at T9 during the low flow season (Figure S1).

The [Cl^−^] in mainstream ranged from 4.67 to 121.4 mg·L^−1^ (mean = 75.1 ± 29.8 mg ·L^−1^, n = 26) during the high flow season (Figure S1). High [Cl^−^] were detected in some of tributaries which ranged from 19.96 to 584.4 mg·L^−1^ (mean = 169.1 ± 193.0 mg ·L^−1^, n = 13) during the high flow season. Almost all samples had higher [Cl^−^] in the low flow season than that in the high flow season (Figure S1).

### Monthly monitoring of nitrogen compound concentrations

**Figure 3:**
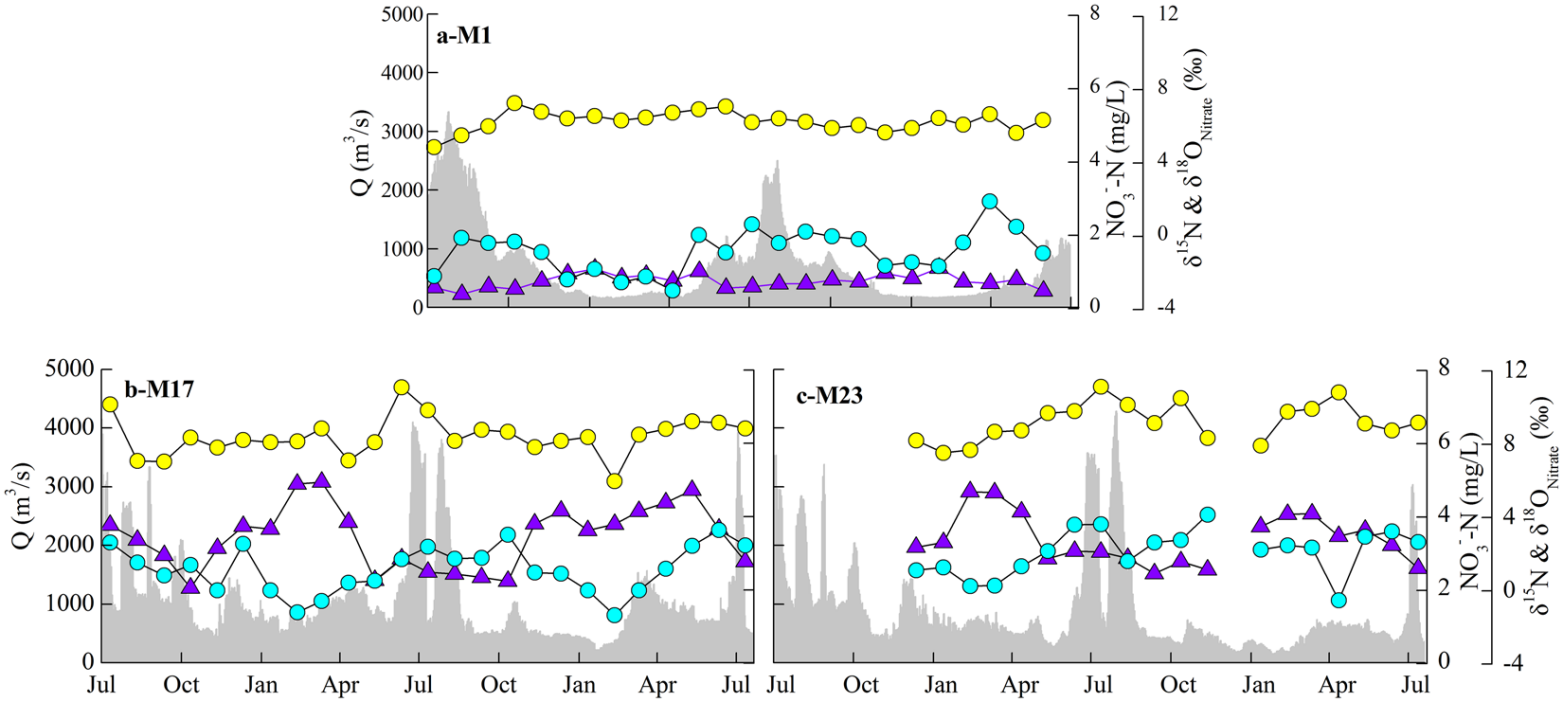
The variations of nitrate characteristics in monthly samples (symbols are same to Fig. 2).

The TDN exhibited variation between the three chosen monthly monitoring sites. The [TDN] at M1 (0.59 to 1.7 mg·L^−1^, mean = 0.95 ± 0.30 mg·L^−1^, and n = 24) was lower than that at the other two sites (one-tailed t-test, p < 0.0001, α = 0.05) (Figure S2). The [TDN] at M17 ranged from 2.4 to 5.8 mg·L^−1^ (Figure S2), with a mean value of 4.0 ± 0.93 mg·L^−1^(n = 25). The [TDN] at site M23 ranged from 3.0 to 5.6 mg·L^−1^ (Figure S2), with a mean value of 4.0 ± 0.75 mg·L^−1^(n = 19). Furthermore, up to 80% of the samples at M17 and M23, and 54% of samples at M1 have a [NO_3_^−^–N]/[TDN] ratio of greater than 80%, indicating that nitrate represented a substantial proportion of the TDN. The [NO_3_^−^–N] at the M17 and M23 sites was three to four times higher than that at M1 sites. Three samples at M17 had [NO_2_^−^–N] of greater than 0.30 mg·L^−1^ during the low flow season. The [TDN] also exhibited temporal variation from 2012 to 2014, with higher concentrations observed during the low flow season at all three sites. The [NO_3_^−^–N] was higher during the dry seasons (from November to April) than in the wet season because of reduction in water fluxes and a relatively high load of nitrogen from anthropogenic inputs (Fig. 3), in agreement with the previous studies^21^.

The flux of N at sites monitored monthly was estimated using the concentration of TDN (C) and water discharge (Q) with the equation: F = C × Q. The average calculated TDN flux were 1.77 × 10^5^ ston/year at M1, 12.7 × 10^5^ ton/year at M17 and 11.0 × 10^5^ ton/year at M23 based on the above equation.

### Long-term variation of N species in the Yellow River

**Figure 4:**
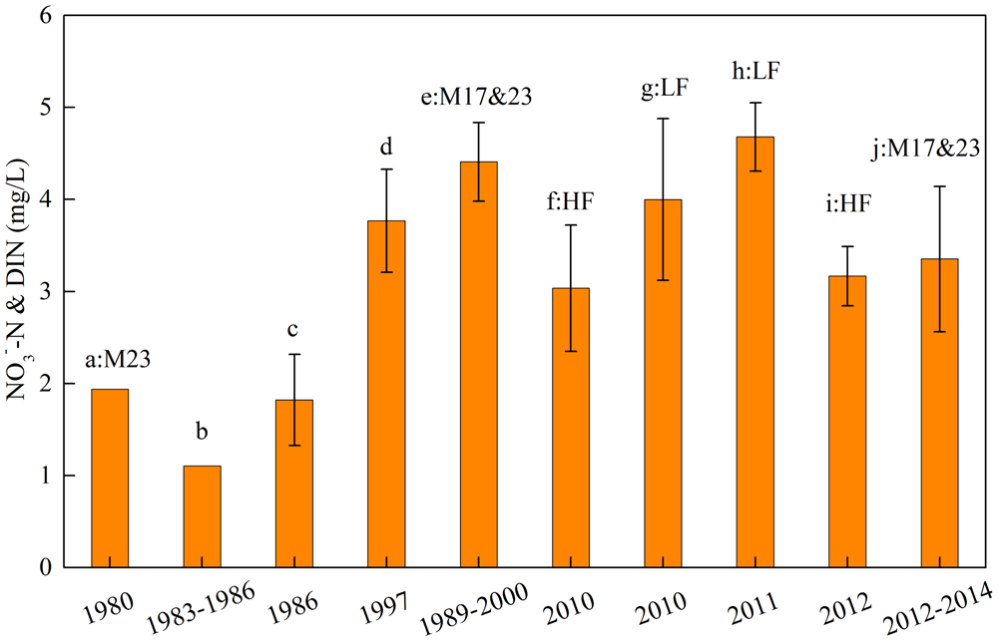
[NO_3_^−^–N] (mean ± sd) in the Yellow River from Tongguan (M13) to the downstream in different years. The branch gives the range of the data, and the histogram gives the average value. (**a**) Samples from Luokou station (M23) during 1980 (n = 24)^22^; (**b**) Samples from lower part of river from November 1983 to August 1986^23^; (**c**)Samples at August 1986 (n = 5)^24^; (**d**) Annual average concentration of NO_3_^−^ –N in the seven mainstream stations during 1997^17^; (**e**) Average concentration of DIN in the two mainstream stations (M17 and M23) from 1989 to 2000^25^; (**f**) Samples in the high flow season in this study (n = 14); (**g**) Samples in the low flow season in this study (n = 14); (**h**) Samples collected from Tongdaoguai (M9) to Lijin (M26) during October 2011(n = 13)^16^; (**i**) Sample in the high flow season during 2012 (n = 9)^26^; (j) Average concentration of NO_3_^−^ –N in two mainstream stations (M17 and M23) from 2012 to 2014 in this study.

As illustrated in Fig. 4, the [NO_3_^−^–N] or dissolved inorganic nitrogen concentration ([DIN]) has increased over the past thirty years. For example, the average [NO_3_^−^–N] at Luokou station (M23) had increased from 1.94 mg·L^−1^ (n = 24) in the 1980s to 3.4 ± 0.7 mg·L^−1^ (n = 19) during 2012 to 2014, which indicates that the [NO_3_^−^–N] has increased by approximately two-fold during the past thirty years. High [NO_3_^−^–N] was also found in the Yellow River during the low flow season in 2011 (Fig. 4). Results showed a relatively constant [NO_3_^−^–N] (approximately 4.0 mg·L^−1^) in the middle and lower reaches of the YR in recent years. Although an increasing trend in [NO_3_^−^–N] is observed over the past three decades, [NO_3_^−^–N] during the period 2012 to 2014 was lower than the average [NO_3_^−^–N] during 1989 to 2000.

**Figure 5:**
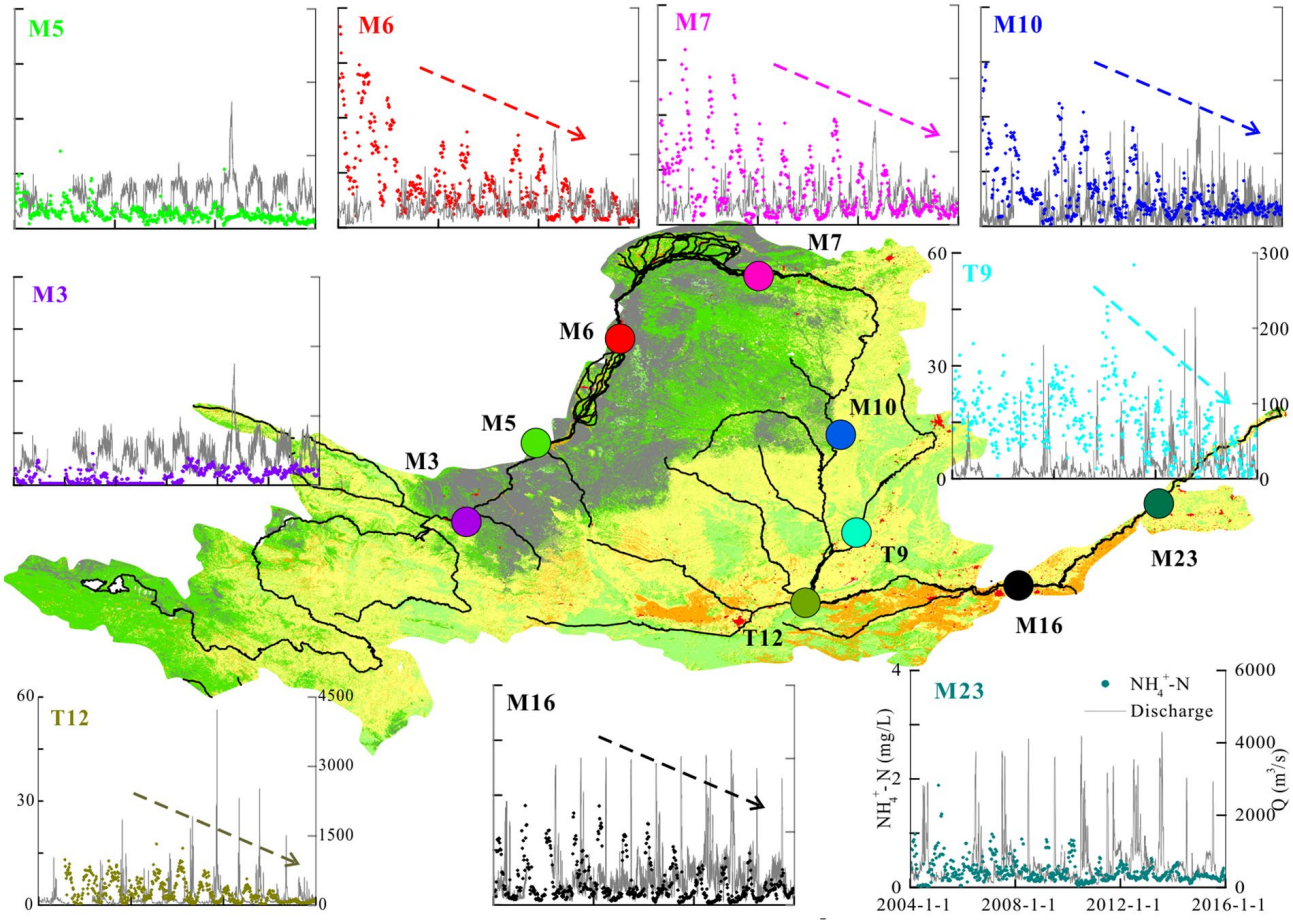
The weekly variations of [NH_4_^+^-N] at the main stations of Yellow River and two tributaries during 2004 to 2015. The data was obtained from Data Center in Ministry of Environmental Protection of the People’s Republic of China (http://datacenter.mep.gov.cn/). The left y-axis indicates [NH_4_^+^-N] and right y-axis indicates water discharge (Q). The x-axis indicates date from 1/1/2004 to 1/1 2016. The range of y-axis is same for all mainstream sites and x-axis is same for all sites. Map produced using ArcGIS software 9.3 (http://www.arcgis.com/features/index.html).

Figure 5 shows the long-term variation of [NH_4_^+^–N] during the period 2004–2015 at nine stations. Significant spatial and temporal variation is observed in the long-term monitoring data. The lowest [NH_4_^+^–N] in the water sample was found at M3 station located in the upper reaches among all nine monitoring sites. A gradual increase occurs from station M3 to station M5, and a three-fold increase is observed between stations M5 and M6. In the middle reaches, [NH_4_^+^–N] decreased between stations M7 and M16.

The [NH_4_^+^–N] in two major tributaries ranged from 0.19 to 60.7 mg·L^−1^ at T9 and from 0.14 to 17.6 mg·L^−1^ at T12, with mean values of 14.4 mg·L^−1^ and 3.71 mg·L^−1^, respectively, which are quite high concentrations for surface water. The values of [NH_4_^+^–N] at T9 and T12 are higher than that of the other stations located in the mainstream (Table S1). Additionally, there is significant seasonal variation in [NH_4_^+^–N] at these sites, with an increase observed from October to December and a decrease until April of the next year. The discharge between 2011 and 2015 did not change greatly at these sites (Fig. 5), but the maximum and average of [NH_4_^+^–N] decreased at most stations between 2011 and 2015, which suggests that environmental protection measures have played an important role in water quality improvement in recent years.

### Seasonal variation in isotopic composition of nitrate

The δ^15^N_nitrate_ values ranged from 7.3‰ to 13.7‰ with a mean value of 8.8 ± 1.6‰ (n = 22) during the low flow season and from 0.1‰ to 12.2‰ with a mean value of 6.9 ± 2.1‰ (n = 39) during the high flow season, respectively. The δ^15^N_nitrate_ values were higher during the low flow season than those during the high flow season (one-tailed t-test, p < 0.0001, α = 0.05). Tributary samples have lower average δ^15^N values (6.0‰, n = 13) than mainstream samples (7.3‰, n = 26) during the high flow season. Samples from the middle reaches had a higher average δ^15^N_nitrate_ value (8.0‰, n = 9) than that in the upper reaches (6.7‰, n = 9) and lower reaches (7.3‰, n = 8) during the high flow season. However, the average δ^15^N_nitrate_ value of samples in the middle reaches was lower than that in the lower reaches (7.8 ± 0.5‰ compared to 8.7 ± 0.5‰) during the low flow season (Fig. 2). The δ^18^O_nitrate_ value did not show significant variation between the low and high flow seasons, ranging between −3.6–6.4‰ in the high flow season and −5.0–7.9‰ in the low flow season, respectively. The mainstream water samples generally exhibited narrow variation in nitrate isotopic values, with the exception of several samples during the low flow season. However, the δ^15^N_nitrate_ values in the tributaries during the low flow season varied significantly (Figs. 1 and 2).

Rain samples collected from the upper reaches and middle reaches yielded δ^15^N_nitrate_ of −5.5‰ and −3.3‰, and δ^18^O_nitrate_ of 52.7‰ and 68.1‰, respectively, during the high flow season. One snow sample exhibited higher isotope values than the rain samples, with δ^15^N_nitrate_ = 10.1‰, and δ^18^O_nitrate_ = 73.1‰ (Table S2). Two types of nitrogen fertilizers were collected from the lower reaches of the YR with δ^15^N = −0.1‰ for urea and −0.4‰ for di-ammonium phosphate (Table S2).

### Monthly monitoring of nitrate isotopes

The nitrate isotopic values in water samples at M1 ranged from 4.9‰ to 7.3‰ for δ^15^N, with an average value of 6.3 ± 0.5‰ (n = 24), and from −3.0‰ to 1.9‰ for δ^18^O_nitrate_, with an average value of −0.8 ± 1.2‰ (n = 25). The δ^15^N_nitrate_ values varied from 6.0‰ to 11.1‰ in the samples at M17 and from 7.5‰ to 11.1‰ in the samples at M23 (n = 19), showing a wider nitrogen isotope range relative to that of the samples at M1. The δ^18^O_nitrate_ values ranged from −1.3‰ to 3.3‰ at M17 and −0.5‰ to 4.2‰ at M23 (Fig. 3).

## Discussion

### The spatial variation of N species in the Yellow River Basin during 2010 and 2011

The upper section of the upper reaches (upstream of site M3) constitute 28% of the entire drainage area of the YR, contributing more than 56% of discharge^14^. High concentrations of [Cl^−^] in the municipal sewage and contaminated groundwater has previously been observed, which indicates that most of the Cl^−^ was mainly of anthropogenic origin^11, 27^. Two tributaries (T1 and T2) and three mainstream samples (M1–M3) in the upper reaches (UR), with low population density (9.5 person/km^2^)^28^, had relative low [Cl^−^] and [NO_3_^−^–N] for these five sites, which amounted to one third of those from other mainstream sites. This result suggests minimal contamination from human activities in this section of the YR. The degree of influence of industrial and agricultural activities on water quality gradually increased after site M3, such as the polluted tributaries of T3 and T4. A heavy load of Cl^−^ and NO_3_^−^–N in river water samples was also detected in the tributaries of the middle reaches (TMR). Ningxia Plain and Hetao Plain are important agricultural districts, where YR water is diverted for irrigation of farmland by a drainage network (shown in Fig. 1 between M5 and M9). Water returning to the YR from these farming regions through drainage channels and groundwater connected with farmland may be a source of nutrients derived from fertilizers and other agricultural sources. Previous studies have calculated that greater than 50% of water diverted for irrigation in these districts returns to the YR, resulting in a significant increase in the concentration of nitrogenous compounds^14, 28, 29^.

The concentration of nitrogen increased between M12 and M13, following the input of two major tributaries (T9, Fen River and T12, Wei River) and indicating high nitrogen concentrations in these tributaries. A significant decrease in [NO_3_^−^–N] was observed between two water conservancy projects, Sanmen Valley Reservoir (near M14) and Xiaolangdi Reservoir (near M15), where water is retained for prolonged periods (Fig. 2). Although the lower reaches (LR) have a high population density (4717 person/km^2^) and developed economy^28^, [NO_3_^−^–N] showed only a slight increase from 2.9 to 3.5 mg·L^−1^ between sites M19 and M26, which may be related to the small number of tributaries entering the main body of the YR. The primary inputs in this section are point sources from industry and domestic waste in city and urban areas.

### Monthly variation of N species at monitoring sites during 2012 to 2014

The monthly trend in NO_3_^−^–N differs between the two years over which continuous monthly data was collected (Fig. 3), which suggested that nitrate concentration was determined by a variety of factors. The estimation of N flux (see results) also indicates that the tributary river between M1 and M17 contributes approximately 86% nitrogen load. Both the concentration and N flux data suggest that the output of N from the YR is mainly derived from the lower section of the upper reaches (M3 to M9) and middle reaches.

The significant seasonal variation in precipitation and agricultural activities may affect riverine nitrogen sources and budget. There are two water discharge peaks, from March to May and from May to October (Fig. 3). The low [TDN] is likely to be due to dilution by snowmelt during the first peak and precipitation during the second. This period is otherwise characterised by high nitrogen input from agricultural activities; the nitrogen flux increased from both point and diffuse sources. The [NO_3_^−^–N] was generally higher at M23 during the second peak than that at M17, suggesting that the relatively constant point sources are more likely to be responsible for the nitrogen concentrations observed in this reach of the YR (Fig. 3).

### Spatial and temporal variation of nitrate sources in the Yellow River

**Figure 6:**
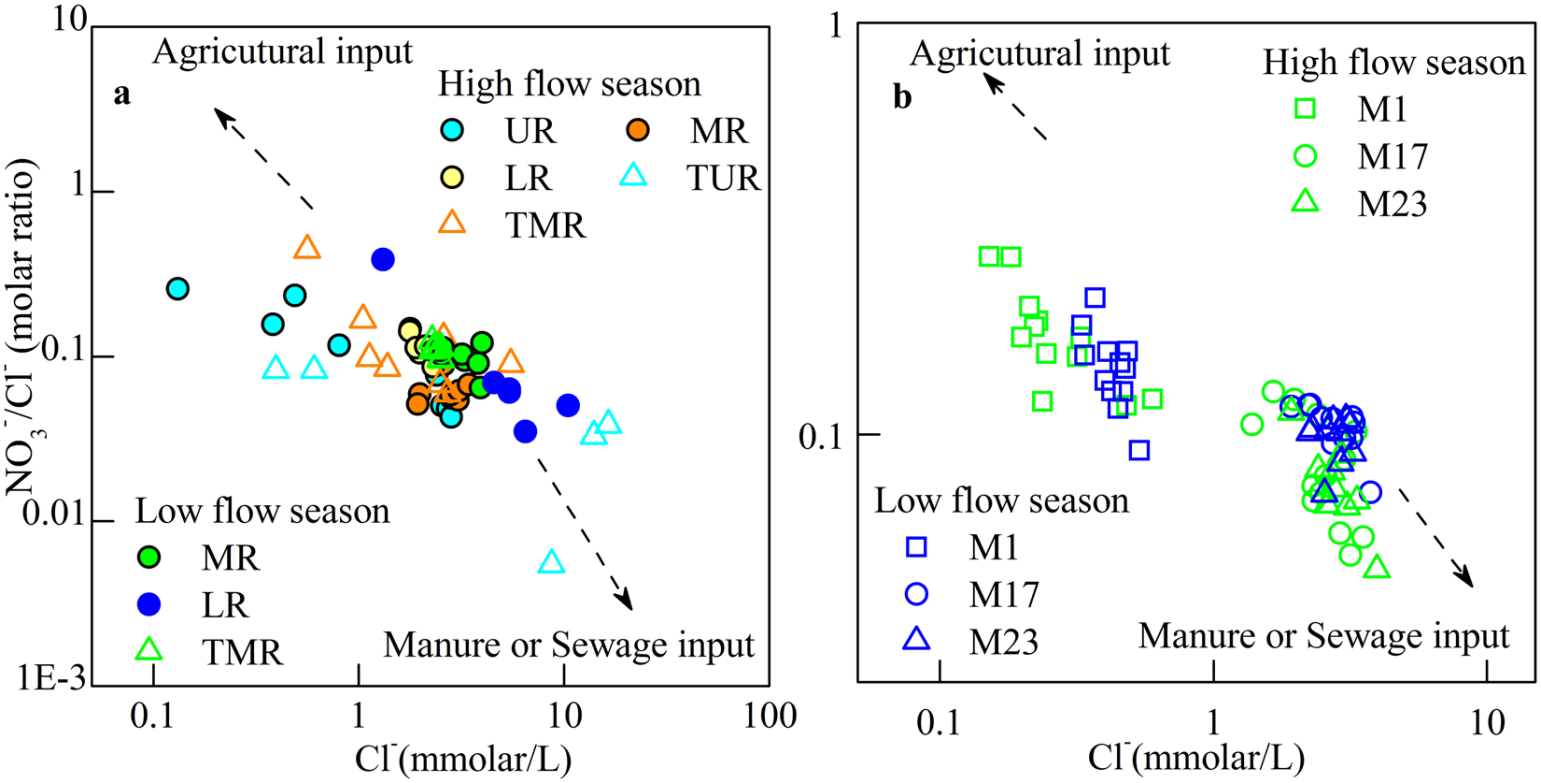
(**a**) The relationship between [Cl^−^] and [NO_3_^−^]/[Cl^−^] for spatial data during the high and low season, YR indicates Yellow River; (**b**) The relationship between [Cl^−^] and [NO_3_^−^]/[Cl^−^] for time series data at three selected sites. UR stands for mainstream samples at upper reaches of YR. MR means mainstream samples at middle reach of YR. LR means mainstream samples at lower reach of YR. TUR means tributaries at UR. TMR means tributaries at MR.

The ratio of [NO_3_^−^]/[Cl^−^] can be used as an indicator of mixing or biological processes affecting nitrate distribution in the watershed, due to the conservative chemical behavior of chloride^10, 11^. Generally, chemical fertilizer contain high concentrations of NO_3_^−^ contents and has a high ratio of [NO_3_^−^]/[Cl^−^], and sewage waste has high [Cl^−^] content and a low ratio of [NO_3_^−^]/[Cl^−^]^11, 27^. Six upstream samples (Fig. 6a) exhibited low [Cl^−^] content and similarly δ^15^N, suggesting a constant natural nitrogen source. The [Cl^−^] in the mainstream samples increased from M5, and the molar ratio of [NO_3_^−^]/[Cl^−^] decreased, which suggested the contribution of sewage waste is increasing, especially during the low flow season. Although the [Cl^−^] and [NO_3_^−^]/[Cl^−^] in tributaries exhibited more variation than mainstream samples during the high flow season (Fig. 6a), the small variation in the mainstream values indicates that sufficient mixing occurs in the mainstream to homogenize the signature. The monthly monitored sites (Fig. 6b) displayed significant variation from the low [Cl^−^] and molar ratio of [NO_3_^−^]/[Cl^−^] at M1, which is similar to the water samples from the upstream sites (Fig. 6a).

**Figure 7:**
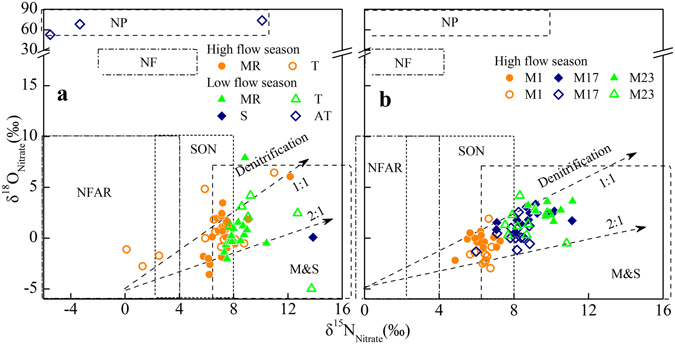
Relationship between δ^15^N_nitrate_ and δ^18^O_nitrate_ values during 2010 to 2011 (**a**) and at three monthly sites during 2012 to 2014 (**b**). MR indicates main reaches samples. T stands for tributaries samples. S stands sewage waste sample. AT stands for atmospheric precipitation samples. Other abbreviations for sources were introduced in the text.

The isotopic composition of various nitrogen sources in the study area is shown in Table S2. The content of SON in soil profiles in Loess Plateau decreased and δ^15^N_SON_ increased from top to bottom, and ranged from 1.9 to 8.2‰ (n = 95) with low isotope values in the topsoil of the Loess Plateau (1.9 to 5.1‰, n = 5)^30^. The major forms of N in sewage and hydrolysis fertilizers are NH_4_^+^ (NH_3_) and organic N, which are subsequently converted into nitrate or N_2_ by nitrification, volatilization and denitrification. Nitrate derived from sewage with a higher δ^15^N value than that of raw material is usually characterized by δ^15^N between 7‰ and 25‰^8, 27^. The δ^18^O_nitrate_ values of nitrate derived from nitrification of ammonium from urea fertilizers and rain (NFAR) (Fig. 7) are related to the oxygen isotopic composition of O_2_ and H_2_O in the surrounding environment with a chemical stoichiometric ratio of 1:2. The δ^18^O_water_ ranged from −10.7 to −7.6‰ in all water samples except for one sample (T5) with high δ^18^O_water_ (−3.0‰), which was published in our previous study for the spatial sites^31^. Consequently, nitrate derived from microbial nitrification has δ^18^O_nitrate_ values ranging from 0.7 to 2.8‰. However, several factors would influence the δ^18^O_nitrate_ values during nitrification, such as O exchange between NO_2_^−^ and H_2_O and the diffusion of oxygen, which may lead to a ratio of oxygen atoms from water and O_2_ of greater than 2:1 during nitrification^7, 32–34^. In this study, more than 50% of water samples had δ^18^O_nitrate_ values lower than 0.7‰, suggesting nitrification occurred under low oxygen conditions. Under these anoxic condition, oxygen atoms from water may be a more important source than free oxygen during nitrification. Therefore, the sewage and riverine nitrate with low δ^18^O_nitrate_ values would be primarily impacted by the nitrification process. Nitrate fertilizer (NF) formed from atmospheric oxygen has a distinctive δ^18^O value (18 to 22‰), which is same to the δ^18^O_air_ (23.5‰) (Fig. 7)^7^.

Nitrate derived from the atmosphere precipitation (NP) via photochemical reactions involves compounds enriched in^18^O and thus has higher δ^18^O_nitrate_ values than other nitrate sources (Fig. 7)^7^. To estimate the contribution of atmospheric deposition to riverine nitrate concentrations, a mixing-model based on mass balance equations was used (Supplementary Equations). The average δ^18^O_nitrate_ value from atmospheric deposition was assumed to be 64.8‰ and the δ^18^O_water_ during nitrification was calculated to be 0.7 to 2.8‰. If the contribution from atmospheric deposition is 10%, the δ^18^O_nitrate_ value from mixing these two sources would range from 7.1‰ to 9.0‰, based on the mixing model. The δ^18^O_nitrate_ values were between −3.0‰ and 1.9‰, which indicates that the contribution from atmospheric precipitation was limited. Therefore, atmospheric nitrate is not a major source for the YR, which is in agreement with previous studies based on limited sampling campaigns^16, 35^.

The riverine nitrate in the Yellow River might be impacted by various factors in different zone due to sparse distribution of dual isotopic characteristics (Fig. 7a). The riverine nitrate in tributaries in the upper reaches would be derived from the nitrification of manure and sewage waste (M&S) as well as chemical fertilizers, based on the dual isotopic pattern of nitrate. The Fen River and Wei River entered the mainstream with high NO_3_^−^–N, and low δ^15^N_nitrate_ (1.3‰, 2.5‰, respectively) and δ^18^O_nitrate_ (−2.8‰, −1.7‰, respectively) values, indicating that chemical fertilizer was the major source for these two rivers, similar to the results of other study^16^. Although these two river catchments had high population densities, the abundant rainfall during the high flow season and routine application of fertilizers may lead to a major contribution from chemical fertilizers. The isotopic values of these two tributaries changed significantly between the two seasons, with high isotope values in the low flow season. These two major tributaries had high NH_4_^+^–N during both two seasons, but the samples had higher [NH_4_^+^–N] and δ^15^N values during the low flow season than that during the high flow season, indicating that manure and sewage waste were the major source instead of nitrogen from agriculture. The high DON and nitrate isotopic composition (δ^15^N: 7.7–12.7‰; δ^18^O_nitrate_: 1.2–4.2‰) of the water samples collected from other tributaries showed that manure and sewage waste were major contributors to riverine nitrate during this period.

The small monthly variation of dual isotopes (M1, Fig. 6b) suggests that nitrate sources were constant in the headwaters. The catchment area for M1 is the alpine zone, where more than 60% of land use is natural grassland^28^. Therefore, SON might be a more important nitrate source. Nitrate concentration increased slightly during the low flow season, when the level of microbial activity is lower due to low temperature conditions, and the average nitrate concentration (0.75 ± 0.19 mg·L^−1^) suggests the presence of other nitrogen sources with high nitrogen concentrations and similar δ^15^N values (4.9 to 7.3‰). Animal manure produced by free grazing of livestock in this region may be responsible for the variation in nitrate concentrations. Therefore, nitrate in the upper reaches (M1) is mainly derived from mineralization of SON and animal manure. The increased [NH_4_^+^–N] at long-term monitoring sites located in the lower section of the upper reaches (M3–M9), suggests an anthropogenic source of N, such as nearby urban areas. Although fertilizer application and precipitation are limited during the low flow season, flood irrigation would allow nitrogenous fertilizers to infiltrate the groundwater, resulting in nutrient discharge into rivers^36^. Therefore, sewage waste and chemical fertilizer may be responsible for the high [NH_4_^+^–N]. Riverine nitrate in the middle reaches exhibited a wider range in δ^15^N than that in the upper and lower reaches, which suggests that multiple nitrogen sources contribute to the middle reaches across different seasons. The distribution of nitrate isotope values in the middle reaches indicates that nitrate sourced in the upper reaches of the YR and the tributaries located in the middle reaches are impacted by multiple sources, including chemical fertilizers, SON, manure and sewage waste. The higher isotope values of nitrate at M17 and M23 than at M1 indicate the addition of further sources between these sites that are enriched in^15^N, such as manure and sewage waste, or that riverine nitrate is affected by isotopic fractionation by denitrification. The [NO_3_^−^–N] is higher in the lower reaches, but the isotopic values showed no significant change, indicating similar sources to those encountered in the middle reaches.

Overall, it can be concluded that the tributaries in the middle reaches were polluted by manure or sewage waste and this dominated the nitrate pool. Nitrate in samples from the upper reaches would be from SON, while agricultural sources, chemical fertilizer and manure waste dominate in the lower section of the upper reaches. The middle reaches are influenced by multiple sources and may be affected by the significant variations observed in tributaries during high flow season. The concentration of nitrogen and difference in isotopic signature of nitrate indicates that manure and sewage waste was a larger contributor to the Yellow River Basin during the low flow season.

### Major transformations in the riverine nitrogen cycle

The main processes that control nitrogen dynamics in ecosystems commonly result in an increase in the δ^15^N of the substrate, unless the reactions go to completion^7^, such as ammonia losses from organic matter within manure, which may result in δ^15^N values higher than 25‰^27^. The middle and lower reaches of the basin drained a greater area of agricultural land and were subject to fertilizer application during the high flow season. In theory, the nitrogen isotope values during this season may have low values close to 0‰. However, the measured compositions were greater than the theoretical values, which indicates that volatilisation may occur and enrich the heavy isotopes. Xing and Zhu (2002) reported that the loss of N through volatilization of urea was 22% in agricultural land based on mass budget^37^. The volatilization process follows the Rayleigh distillation equation with an overall fractionation effect of approximately −20‰^38^. Based on the conversion factors, the Rayleigh fractionation equation, and δ^15^N values of nitrogenous fertilizers collected within the basin, the δ^15^N of the residual fertilizer nitrate would be 4.6‰, which is close to that observed in tributary samples from the middle reaches.

Nitrification is a multi-step oxidation process mediated by several different autotrophic organisms. Several samples showed δ^18^O_nitrate_ values higher than the theoretical δ^18^O_nitrate_ value (2.8‰), indicating that nitrification was the major transformation in the nitrogen cycle. Additionally, the long-term observation of ammonium showed high concentration in the low flow season and low concentrations during the high flow season with warm temperature, indicating that nitrification was the dominant process controlling the temporal variation of ammonium. Meanwhile, the increasing trends of oxygen isotopic values in water and oxygen isotopic values of nitrate along the flow path suggest nitrification played an important role in production of nitrate^31^.

However, nitrate isotopic values would increase during other biological processes in the riverine system, such as algae uptake and denitrification^7, 39^. The decreased concentration and highest dual isotopes of nitrate in the mainstream of the Xiaolangdi water conservancy project (M15, Fig. 2) suggested riverine nitrate may be affected by algae assimilation uptake and denitrification. As indicated by Fig. 7b, a positive relationship between δ^15^N and δ^18^O_nitrate_ was observed (δ^18^O_nitrate_ = 0.79 × δ^15^N-5.47, R^2^ = 0.49, *p* < 0.0001), particularly for samples collected in the high flow season (δ^18^O_nitrate_ = 0.73 × δ^15^N-4.53, R^2^ = 0.70, *p* < 0.0001). The decreased nitrate concentration and increased δ^18^O_nitrate_ value at M1 and M17 sites during the high flow season (Fig. 3) may reflect dilution, an increased contribution from atmospheric nitrate, and denitrification. Liu *et al*.^16^ reported that the nitrate transformation might be mainly influenced by microbial denitrification as the high concentration of suspended sediment makes it difficult for algae/phytoplankton to grow^16^. High concentration of suspended sediment can accelerate rates of denitrification, even in aerobic waters^16, 18^. Moreover, denitrification could remove N in riverine water column at equivalent or higher rates than headwaters^40^. Therefore, the increased δ^18^O_nitrate_ values during the high flow season indicate that nitrate isotopes would be affected by denitrification in this studied river.

### Nitrate characteristics in the Yellow River compared to other large rivers worldwide

Table 1 lists [NO_3_^−^–N] and isotopic composition of nitrate in large rivers obtained at long-term monitoring sites, which collect biweekly or monthly samples for more than one year. Due to the influence of snowmelt-derived water, the nitrate characteristics show lower [NO_3_^−^–N] and δ^15^N, and higher δ^18^O_nitrate_ in Saint Lawrence River than in the other four large rivers^9, 41, 42^. Although the upper reaches of the YR also includes the snow region, the nitrate characteristics at the M17 site (Table 1) appear to differ from those of the Saint Lawrence River. Among the four large rivers worldwide, the [NO_3_^−^–N] is the highest in the YR. Nitrate isotopes are similar with those of two German large rivers that are highly impacted by human activities, with higher δ^15^N and lower δ^18^O_nitrate_ than that of the Saint Lawrence River and Mississippi River^9, 41, 42^.

**Table 1:** Nitrate characteristics for long-term monitoring sites in large rivers.

River	Length	Basin Area	Mean- [NO_3_^−^–N]	Mean-δ^15^N_nitrate_	Mean-δ^18^O_nitrate_	Sample Number
	km	10^5^ km^2^	mg/L	‰	‰	
Mississippi River^42^	6020	32.2	1.45 ± 0.5	7.7 ± 0.3	4.6 ± 0.3	22
Yellow River	5465	7.95	3.4 ± 0.86	8.4 ± 1.1	1.2 ± 1.3	25
Saint Lawrence River^41^	1287	3.00	0.43 ± 0.08	5.7 ± 0.7	5.4 ± 1.4	41
Rhine River^9^	1320	1.85	2.7 ± 0.7	8.2	0.4	24
Elbe River^9^	1165	1.48	2.6 ± 1.4	8.5	1.3	16

These rivers exhibited seasonal variations is nitrate pool composition, such as low NO_3_^−^–N during the high flow season and high δ^15^N during the low flow season, reflecting variation in hydrological and nitrate sources^43, 44^. The high contribution of urban sewage waste may be responsible for the high δ^15^N during the low flow season. Nitrate isotopic composition is closely related to the nitrate sources and water flow in the Illinois River, with isotope values in river samples close to that of tile drain samples during the high flow period and treated wastewater during the low flow period^39^. The difference of nitrate isotopic values among M1, M17 and M23 sites in the present study also suggests that nitrate isotopic composition depends on the sources, as in M1, and the water flow state, as observed in the seasonal variation between flow seasons in M17 and M23.

In this study, the long-term variations of nitrogen compound concentrations suggest nitrate is the dominant nitrogen compound during both flow seasons. The tributaries exhibit high [NO_3_^−^–N] and [NH_4_^+^–N]. Moreover, the [DON] and [NH_4_^+^–N] exhibit seasonal variation, with high concentrations in the low flow season. Nitrogen sources in two major tributaries are different, due to agricultural import during the high flow season and manure and sewage waste in the low flow season. The middle reaches in the YR received nitrate from tributaries with a distinct agricultural signature and urban input. The dual isotopic compositions of nitrate during low flow season indicate an increased contribution of sewage waste. Moreover, the isotopic compositions of nitrate and the hydraulic conditions imply that lower reaches riverine nitrate is mainly derived from sewage waste from cities, particularly domestic and industrial waste. The trends of [NO_3_^−^–N] and [NH_4_^+^–N] observed in monthly samples suggest nitrogen pollution has decreased. The results also suggest that riverine processes affect nitrogen transformation and transport processes, such as nitrification and denitrification. The nitrogen concentrations in the YR are relatively high compared with other large rivers around the world. The urban input of sewage waste and irrigation water should be managed to further reduce the nitrogen load and improve water quality in the Yellow River Basin.

## Methods

### Study Area

The Yellow River (Huang River), located between 32°10′N–41°50′N and 95°53′E–119°10′E, is the world's fifth longest river and the second longest river in China, with a length of 5464 km. The mainstream of the YR is commonly divided into three sections, the upper reaches (M1–M9), middle reaches (M9–M18) and lower reaches (M18–M26) (detail in Table 1 and Fig. 1). The upper reaches can also be divided into two sections, the upper section which is upstream of site M3 and the lower section between sites M4 and M9. An unusually large amount of mud and sand (37.8 kg/m^3^) is discharged into the river from the Loess Plateau located in the middle reaches which constitutes 92% of riverine silts, making it the most sediment-laden river in the world and the spectacle after which it is named^14^. The riverbed in the lower reaches is elevated above the ground surface by more than 10m in some areas, due to the building of levee banks to combat flooding accompanied by continuous sediment deposition due to the extremely high sediment transport from the middle reaches. The abundant hydrodynamic resources distributed in the middle reaches where the elevation of the river drops 890 m make it highly suitable for hydroelectric power generation. Two major water conservancy projects (Sanmen Valley Reservoir near M14 and Xiaolangdi Reservoir near M15) are located in the middle reaches. The two largest tributaries (Wei River and Fen River) of the YR are in the middle reaches.

The Yellow River Basin has a continental monsoon climate and most of the basin belongs to the temperate-arid and semi-arid climates^14, 28^. The three primary agricultural production basins are Ningxia Plain and Hetao Plain, Fen Wei Basin (Fen River and Wei River) and lower reaches. In the agricultural regions, synthetic fertilizers are generally applied twice a year, in July for summer crops and October for winter crops. However, manure is also applied periodically throughout the year. Three large cities, Xi’an, Zhengzhou, and Jinan, each have populations of greater than seven million people, and more than three million people live in urban areas surrounding the mainstream or major tributaries. Dry land area accounts for 80% of the basin area. The cultivated land is mainly in the middle and lower reaches, with 7.2% irrigated agricultural area. The majority of grazing land is located in the upper reaches. Grassland, cropland, wetlands, and urban areas account for 60%, 29.5%, 1.1%, and 6% respectively^28^.

### Sampling campaigns

The seasonal water samples were collected from the mainstream of the YR and its major tributaries in August of 2010 and February of 2011, corresponding to the high and low flow seasons respectively. Water samples comprised 26 mainstream water samples (M1–M26), 13 tributary water samples (T1–T13), and 2 rain water samples during the high flow season. The upper reaches were frozen in winter, resulting in low water discharge into the mainstream. Therefore, water samples were collected from the middle and lower reaches during the low flow season, comprising 22 river samples (16 mainstream and 6 tributaries), one snow sample and one sewage sample. Additionally, three sites (M1, M17 and M23) were selected for monthly sampling from July 2012 to June 2014. Water samples were filtered through 0.45-µm cellulose-acetate filter paper at the field site and collected in 100mL opaque plastic bottles for nutrient and nitrate isotope analysis, and samples for isotopic analysis were refrigerated at 4°C.

### Laboratory analysis

Anion (Cl^−^ and NO_3_^−^) concentrations (mg/L) were determined using a Dionex ion chromatography (IC) system 90 (Dionex Corp., Sunnyvale, CA, USA). The N concentrations (mg/L) of NH_4_^+^, NO_2_^−^, and TDN in the filtered samples were analyzed using an automatic flow analyzer (SKALAR Sans Plus Systems) after the field work. The nitrogen detection limit was 5 μg·L^−1^ for NO_2_^−^–N, 10 μg·L^−1^ for NO_3_^−^–N 10 μg·L^−1^for NH_4_^+^–N, and 20 μg·L^−1^ for TDN. The DON concentration was calculated by subtracting DIN (the sum of NO_3_^−^, NO_2_^−^, and NH_4_^+^ concentrations) from the TDN. Denitrifer bacteria were used to transform the nitrate to nitrous oxide (N_2_O). Isotopic analysis of the N_2_O product was performed using an isotope ratio mass spectrometer after purification using Trace Gas Pre-concentrator unit^45^. For calibration, four international nitrate (USGS-32, USGS-34, USGS-35 and IAEA-N3) and experimental reference materials were treated in an identical manner as the filtered water samples. The analytical precision for the samples analyzed in duplicate was 0.3‰ for δ^15^N and 0.5‰ for δ^18^O of nitrate.

The isotope ratios are reported as delta (δ) expressed in per mil (‰) using the usual delta notation:

1 $${\rm{\delta }}(\textperthousand )=(({R}_{sample}/{R}_{standard})-1)\times 1000$$

where *R* = ^15^N/^14^N or^18^O/^16^O. The^15^N/^14^N reference was N_2_ in air and the^18^O/^16^O reference was Vienna standard mean ocean water.

## Electronic supplementary material

Supplementary information
