# Supplementary material for: Tracing nitrate sources with dual isotopes and long term monitoring of nitrogen species in the Yellow River, China
Source: Sci Rep. 2017 Aug 17;7:8537. doi: 10.1038/s41598-017-08756-7 (PMC5561216; doi:10.1038/s41598-017-08756-7)
Supplement: Supplementary file 1 — Supplementary information [file 41598_2017_8756_MOESM1_ESM.pdf]

Supplementary Information *for*

Tracing nitrate sources with dual isotopes and long term monitoring of nitrogen species in the Yellow River, China

Authors: Fu-Jun Yue, Si-Liang Li*, Cong-Qiang Liu*, Zhi-Qi Zhao, Hu Ding

*Si-Liang Li (siliang.li@tju.edu.cn)

Contents:

1. Supplementary tables
    1.1. Supplementary Table S1
    1.2. Supplementary Table S2
    1.3. Supplementary Table S3
2. Supplementary figures
    2.1. Supplementary Figures S1
    2.2. Supplementary Figures S2
3. Supplementary Equations
4. Supplementary References

1. Supplementary Tables:

1.1 Supplementary Table S1

Table S1 The statistics of weekly [NH_4^+-N] for nine stations

[NH_4^+–N] (mg/L)	M3	M5	M6	M7	M10	M16	M23	T9	T12
Number	620	609	618	619	611	619	617	541	567
Min.	0.02	0.06	0.06	0.01	0.02	0.03	0.01	0.19	0.14
Max.	0.61	1.41	3.66	4.24	2.96	1.81	1.88	60.7	17.6
Ave.	0.17	0.27	0.75	0.64	0.58	0.38	0.28	14.4	3.71
Med.	0.14	0.23	0.55	0.41	0.40	0.24	0.23	14.0	2.58
Std.	0.13	0.15	0.65	0.59	0.50	0.36	0.20	8.59	3.26

1.2 Supplementary Table S2

Table S 2 The isotopic composition of various nitrogen sources.

Endmember	Reference		Study area		
	$\delta^{15}N$	$\delta^{18}O_{nitrate}$	$\delta^{15}N$	$\delta^{18}O_{nitrate}$	number
Nitrate fertilizer[1]	-8~7	17~25			
Reduce fertilizer[1]			-0.3		2
Soil organic nitrogen[1,2]	2~8		3.4[3]		5
Atmospheric nitrate[1,2]	-13~13	25~94	-4.4	60.4	2
Sewage waste[4-6]	4.3~29.9	-4.9~9.4	13.8	0.1	1

1.3 Supplementary Table S3

Table S3 The characteristics of three sections in Yellow River

Stream	Sites	Length	Area	Area proportion	T	Rainfall	Population density[7]
		km	$\times 10^5$ km^2	%	℃	mm	person/km^2
Upstream	M1-M9	3472	4.28	53.8	1–8	390	47.2
Midstream	M9-M18	1206	3.44	43.3	8–14	517	143.0
Downstream	M18-M26	786	0.23	2.9	12–14	651	4717.4

2. Supplementary Figures:

2.1. Supplementary Figure S1

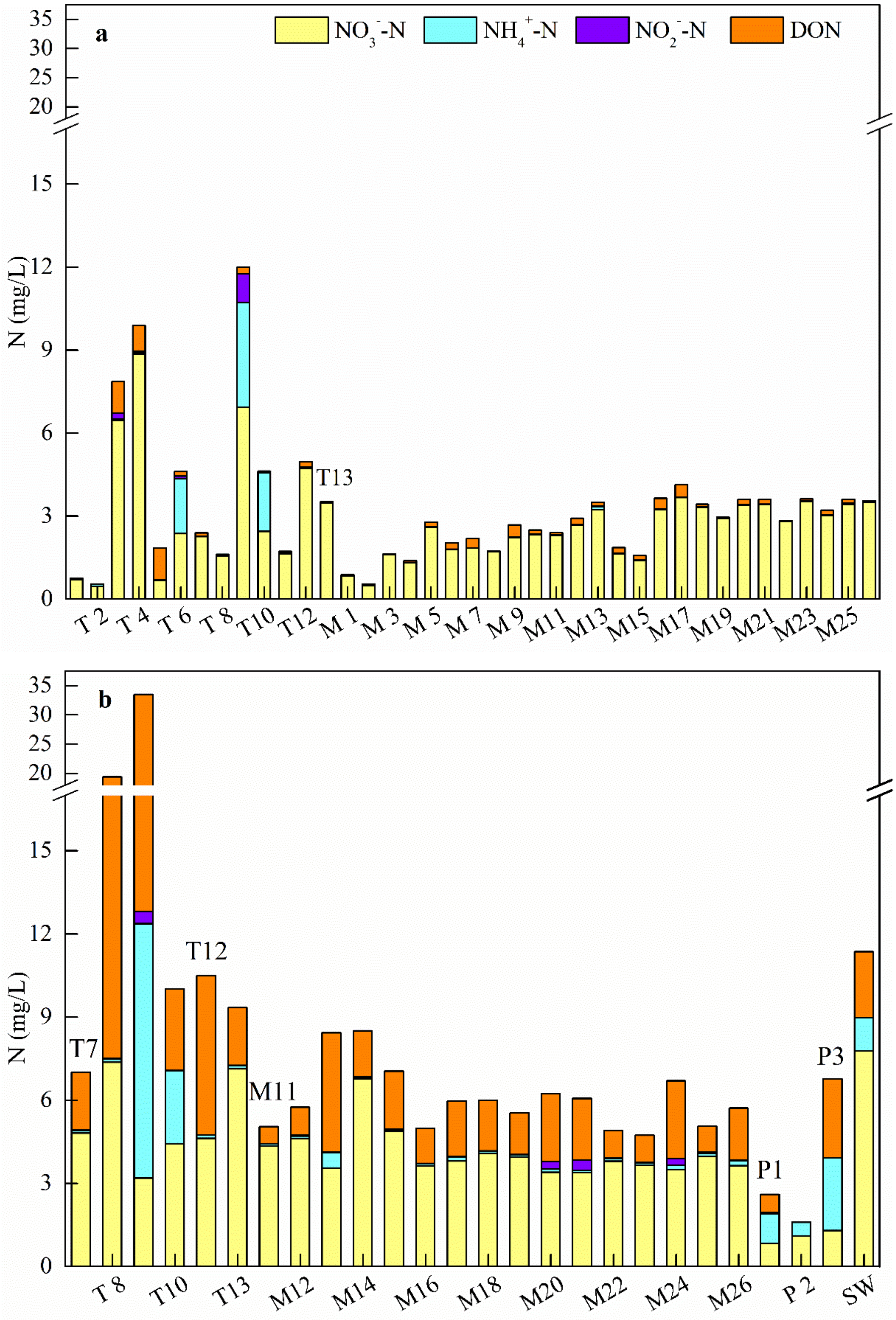

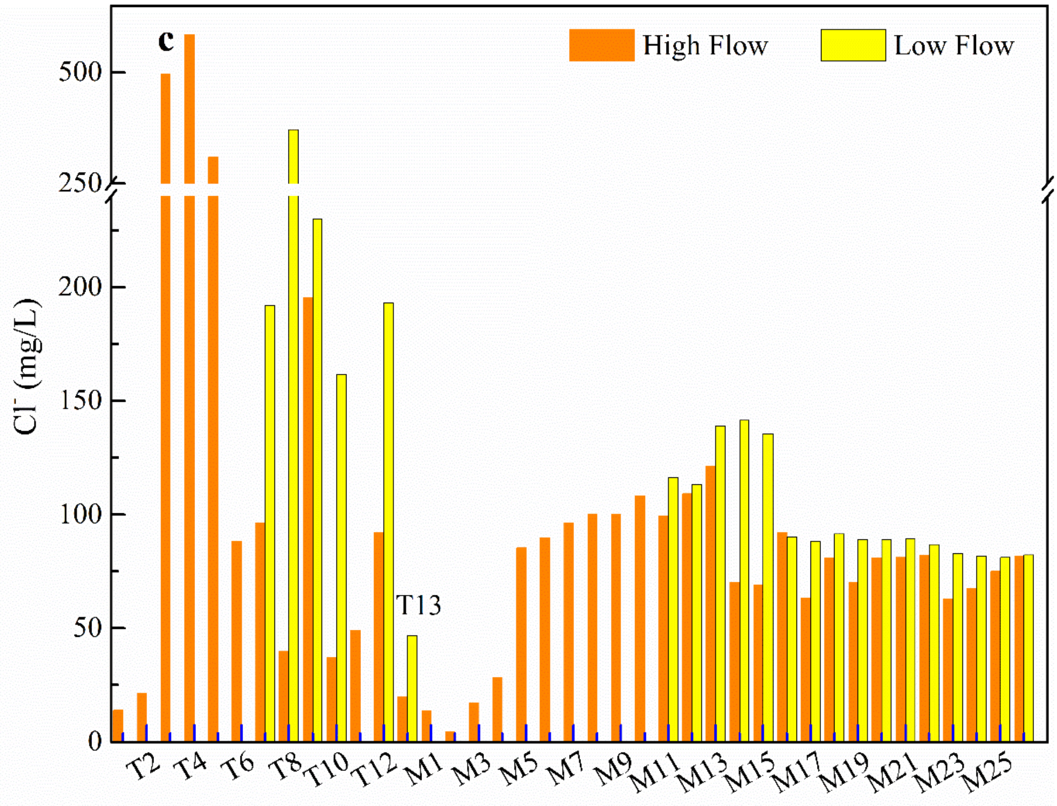

Figure S1. The concentrations of N-species in Yellow River and its tributaries during the high (a) flow season, (b) low flow season. (b included precipitation (P1-P3) and sewage waste (SW) samples) and (c) seasonal Cl^- concentration. Figures were produced using OriginPro 8.5 (http://www.originlab.com/).

2.2 Supplementary Figure S2

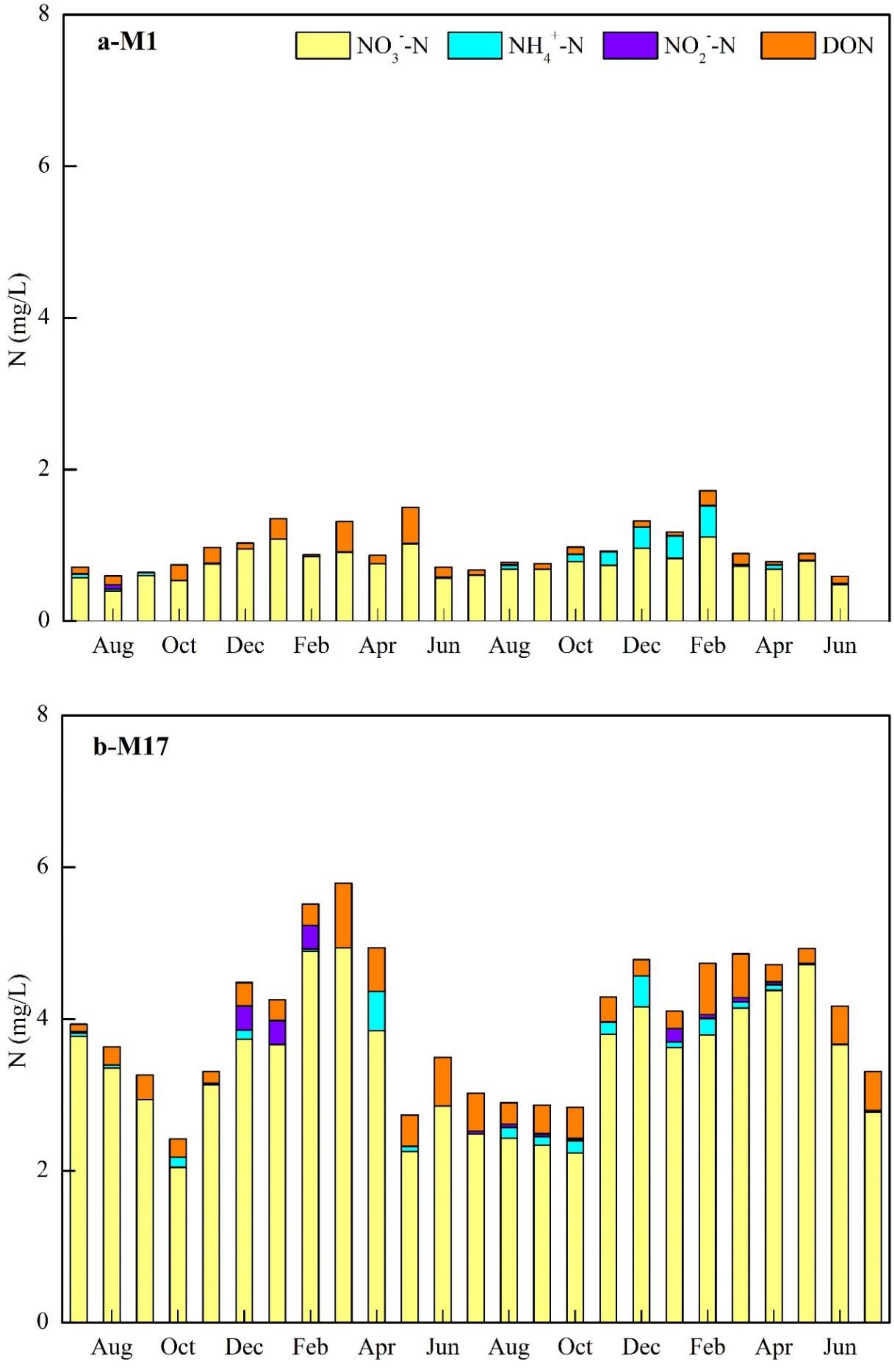

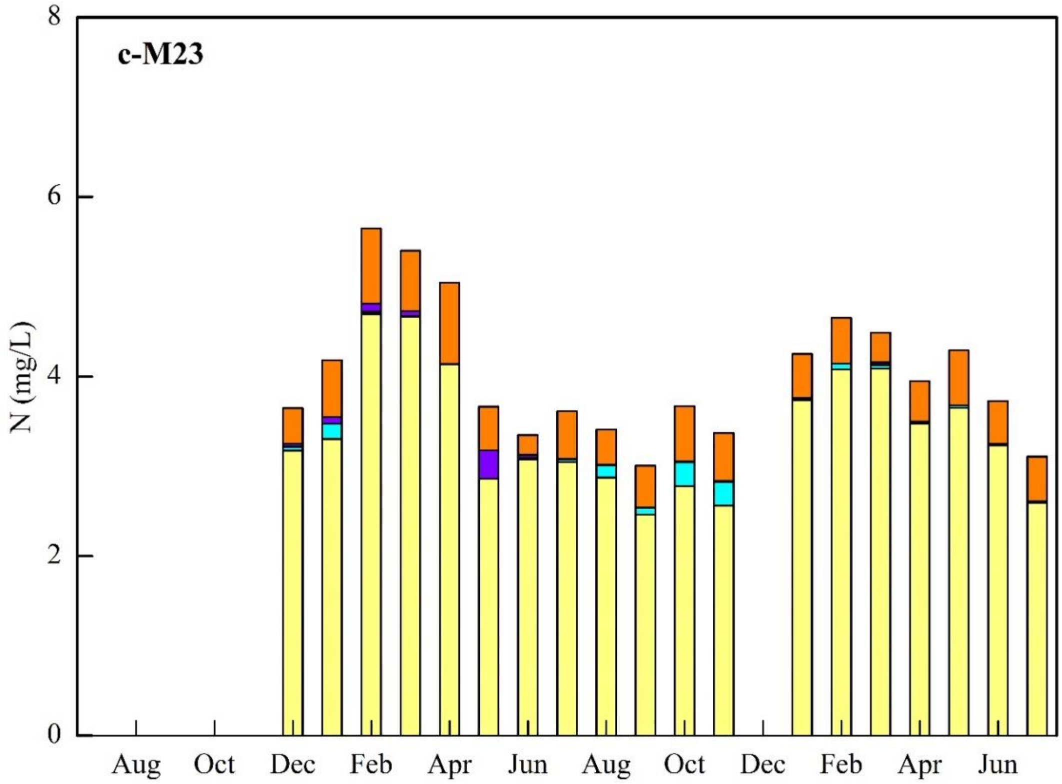

Figure S2 The concentrations of N-species in Yellow River in monthly samples (a) M1, (b) M17 and (c) M23. Figures were produced using OriginPro 8.5 (http://www.originlab.com/).

3 Supplementary Equations

To estimate the contribution of the nitrate sources to the Yellow River a mixing-model based on mass balance equations was used. The model only roughly considers nitrate contributions from only two sampled sources: atmospheric deposition (A) and nitrate originated from nitrification (N). The equations are

$$\delta^{18}O_Y=f_A\times\delta^{18}O_A+f_N\times\delta^{18}O_N \quad (1)$$

$$1=f_A+f_N \quad (2)$$

The subscripts A and N represent: atmospheric deposition (A) and nitrate originated from nitrification (N), Y represents the Yellow River. f is defined as the fraction of the respective source.

4 Supplementary References

1. Kendall, C., Elliott, E. M. & Wankel, S. D. Tracing anthropogenic inputs of nitrogen to ecosystems. *Stable isotopes in ecology and environmental science*, 375-449 (2007).
2. Xue, D. M. *et al.* Present limitations and future prospects of stable isotope methods for nitrate source identification in surface- and groundwater. *Water Res.* **43**, 1159-1170, (2009).
3. Li, L. B. *Isotope geochemistry of soil carbon and nitrogen in typical soil types in Guizhou karstic and Loess Plateau areas* Doctor thesis, University of Chinese Academy of Sciences, (2012).
4. Li, S. L. *et al.* Assessment of the sources of nitrate in the Changjiang River, China using a nitrogen and oxygen isotopic approach. *Environ. Sci. Technol.* **44**, 1573-1578, (2010).
5. Widory, D., Petelet-Giraud, E., Negrel, P. & Ladouche, B. Tracking the sources of nitrate in groundwater using coupled nitrogen and boron isotopes: A synthesis. *Environ. Sci. Technol.* **39**, (2005).
6. Yue, F. J., Li, S. L., Liu, C. Q., Zhao, Z. Q. & Hu, J. Using dual isotopes to evaluate sources and transformation of nitrogen in the Liao River, northeast China. *Appl. Geochem.* **36**, 1-9, (2013).
7. Kusuda, T. *The Yellow River: Water and Life*. (World Scientific, 2010).
